# Comparison of Lateral and Dorsal Recumbency during Endoscope-Assisted Oophorectomy in Mature Pond Sliders (*Trachemys scripta*)

**DOI:** 10.3390/ani10091451

**Published:** 2020-08-19

**Authors:** Edoardo Bardi, Giulia Antolini, Emanuele Lubian, Valerio Bronzo, Stefano Romussi

**Affiliations:** 1 Department of Veterinary Medicine, University of Milan, via dell’Università 6, 26900 Lodi, Italy; giuliaantolini25@gmail.com (G.A.); valerio.bronzo@unimi.it (V.B.); stefano.romussi@unimi.it (S.R.); 2 Veterinary Clinical and Husbandry Centre (CCVZS), University of Milan, via dell’Università 6, 26900 Lodi, Italy; emanuele.lubian@hotmail.com

**Keywords:** chelonians, coelioscopy, endoscopic surgery, oophorectomy, pond slider, *Trachemys scripta*

## Abstract

**Simple Summary:**

Ovariectomy via the prefemoral fossa, with or without endoscopic assistance, is a well-described technique for elective and therapeutic sterilization in chelonians. The choice between lateral and dorsal recumbency is generally left to the surgeon’s preference, with no data supporting an objective superiority of one over the other. In pond sliders, common pets but also an invasive alien species in Europe, we compared two different recumbencies (right lateral with a left fossa approach, and dorsal with a right fossa approach) regarding ease of access to the coelomic cavity, ease of identification of the ovary opposite to the surgical breach, first and second ovary removal times, and total surgical time. No significant difference was found between the two groups for any evaluated parameter. Endoscope-assisted prefemoral ovariectomy in mature pond sliders can be indifferently performed in lateral or dorsal recumbency, depending on the preference of the surgeon, on the animal’s pathophysiologic status, or on the necessity to perform concurrent procedures.

**Abstract:**

Ovariectomy via the prefemoral fossa is a well-described technique for sterilization in chelonians. The choice between lateral and dorsal recumbency is generally left to the surgeon’s preference, with no data supporting an objective superiority of one over the other. Twenty-four sexually mature female pond sliders (*Trachemys scripta*) were enrolled for elective prefemoral endoscope-assisted oophorectomy, and were randomly divided in two groups: 12 animals were placed in right lateral recumbency with a left fossa approach (Group A), and 12 in dorsal recumbency with a right fossa approach (Group B). Scoring systems were applied to assess the ease of access to the coelomic cavity, and the ease of identification of the ovary opposite to the surgical incision. A negative correlation was found between the body weight of the animals and the ease of access to the coelom (*p* = 0.013), making the access easier in smaller animals. No significant difference was found between the two groups in terms of ease of access to the coelomic cavity, first ovary removal time, ease of identification of the second ovary, second ovary removal time, or total surgery time.

## 1. Introduction

Historically, ovariectomy and ovariosalpingectomy in chelonians have been performed to deal with afflictions of the reproductive tract, and more recently, for population control [1,2,3,4]. The increase in requests for elective sterilization in these species is surely, in part, due to the development of minimally invasive techniques such as prefemoral coeliotomy, which allows the surgeon to avoid plastron osteotomy—a much more invasive procedure with a prolonged recovery time and greater possibility of post-operative complications [5,6]. Both elective and therapeutic oophorectomy/ovariosalpingectomy via prefemoral coeliotomy, with or without endoscopic assistance, have been extensively described and documented [2,3,5,6,7,8,9,10]. However, to date, no indication has been given as to which recumbency better serves the purpose of a quicker and more comfortable procedure. Therefore, this decision is made based on the species, the size of the patient, and on the surgeon’s preference [2,6].

The present work aims to investigate whether a recumbency (dorsal vs. lateral) is actually preferable for elective endoscope-assisted prefemoral oophorectomy in mature pond sliders (*Trachemys scripta*).

## 2. Materials and Methods

All the patients were privately owned animals referred to the Lodi Veterinary Teaching Hospital, between May and September 2019, for elective surgical sterilization with written, informed consent signed by the owners. The inclusion criteria consisted of clinically healthy adult females with a body weight (BW) over 900 g, maintained in the area of the Po Valley with correct outdoor management, which was defined as adequate space for swimming and basking, with good water quality, correct nutrition, the presence of nesting sites if they were active egg layers, and natural hibernation being allowed through the years. Before enrollment, a blood sample was collected from each animal from the cervical plexus to investigate packed cell volume (PCV), serum total protein (TP) using a refractometer, and white blood cell count (WBC) using the Natt and Herrik method [11]. For each animal, a dorsoventral X-ray projection was performed. Animals with the presence of eggs, PCV below 20% or over 35%, WBC over 19 × 10^3^/µL, and TP below 1.5 or over 8 g/dL, were excluded from the study [12].

All applicable international, national, and/or institutional guidelines for the care and use of the animals were followed. The protocol for care, handling, and sampling of animals defined in the present study was reviewed and approved by the University of Milan Animal Care and Use Committee (OPBA protocol 44/2020).

A total of 27 animals were selected, aged from 5 to 35 years (median 15 ± 7.7 years), with a BW from 925 to 2200 g (median 1480 ± 382.5 g). All the patients were hospitalized one week prior to surgery and individually housed outdoors; food was withheld 48 h prior to surgery [13]. Each subject was randomly assigned to one recumbency group (Group A: right lateral recumbency with a left prefemoral fossa approach; Group B: dorsal recumbency with a right prefemoral fossa approach). All the procedures were performed by the same experienced surgeon, who had a record of over 20 endoscope-assisted oophorectomies with both techniques in this species prior to this study. For each subject, scoring systems based on those previously published in the literature [13] were modified and applied by the surgeon to assess the ease of access to the coelom (Table 1) and the ease of identification of the ovary opposite to approached fossa (Table 2).

Before induction, an attempt to empty the urinary bladder was made by stimulating the cloaca with cotton tip applicators. Anesthesia was induced by an intramuscular (IM) administration of dexmedetomidine (Dexdomitor, Vetoquinol Italia S.r.l., Bertinoro, Italy) 100 µg/kg, ketamine (Nimatek, Dechra Pharmaceuticals PLC, Bladel, The Netherland) 3 mg/kg, midazolam (Midazolam IBI, Giovanni Lorenzini S.p.A, Aprilia, Italy) 0.5 mg/kg, and alfaxalone (Alfaxan, Jurox Limited, Malvern, UK) 8.5 mg/kg. The animals were intubated with a 1.5 or 2.0 mm uncuffed endotracheal tube, and intermittent positive-pressure ventilation was manually provided (two breaths per minute) administering 100% O_2_ without inhalant anesthetics. This protocol was selected as part of a separate study. The anesthetic parameters monitored were the palpebral and corneal reflexes, limb withdrawal latency, cloacal temperature, and heart rate via ECG [14].

**Figure 1:**
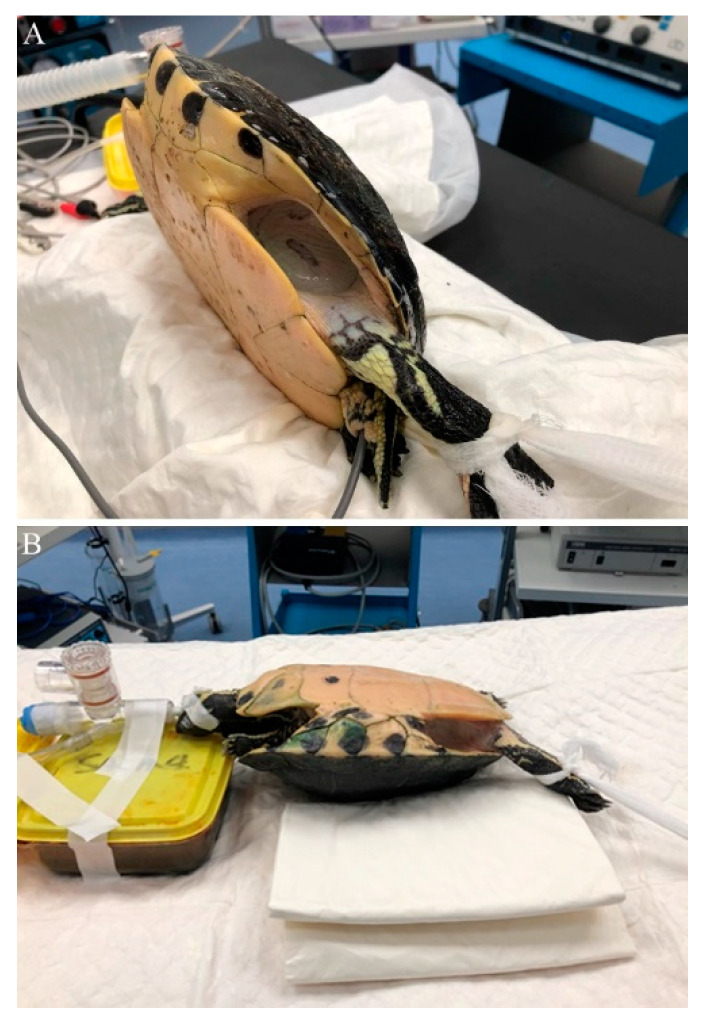
Patients placed in right lateral (**A**) and dorsal (**B**) recumbency, with their hindlimb extended to expose the prefemoral fossa.

Once a surgical anesthetic plane was reached, the animals assigned to Group A were placed in right lateral recumbency inside an empty glovebox lined with a clean puppy pad to prevent rocking movements, and the left hindlimb was extended to better expose the fossa (Figure 1A). The prefemoral fossa and surrounding shell and thigh were aseptically prepared with chlorhexidine solution (Clorexyderm 4%, ICF s.r.l., Palazzo Pignano, Italy), and a fenestrated drape was placed to isolate the surgical site. The animals assigned to Group B were placed in dorsal recumbency; a folded puppy pad was placed underneath to prevent rocking movements, and to create a 30° angle to better expose the right prefemoral fossa (Figure 1B). The ipsilateral hindlimb extension and aseptic dressing of the surgical site were the same as in Group A.

The surgical procedure was performed as previously described [3,5]: a craniocaudal skin incision was made and extended along 75% of the craniocaudal length of the prefemoral fossa, followed by blunt dissection of the subcutaneous tissue. After its exposure and identification, the aponeurosis of the ventral and oblique abdominal muscles, and the coelomic membrane, were pierced with the tip of mosquito forceps to gain access to the coelomic cavity, minimizing the risk of damaging the internal organs.

The coelomic cavity was inspected with a 30°, 2.7 mm × 18 cm rigid endoscope (Hopkins telescope, Karl Storz Endoscopia Italia S.r.l., Verona, Italy), inserted in a 3.5 mm sheath and connected to a xenon light source (Tele Pack XLED TP100, Karl Storz Endoscopia Italia S.r.l., Verona, Italy) to investigate potential abnormalities that did not show up during the health assessment. Animals that showed coelomic alterations were excluded from the study. Cystocentesis was performed if the urinary bladder was distended [6]. After coelomic inspection, the incision on the aponeurosis and on the membrane was extended to match the cutaneous incision, and in those subjects in dorsal recumbency, a ring retractor with elastic stays (Lone Star Retractor System, Cooper Surgical, Origio Italia S.r.l., Roma, Italy) was used to provide exposure.

**Figure 2:**
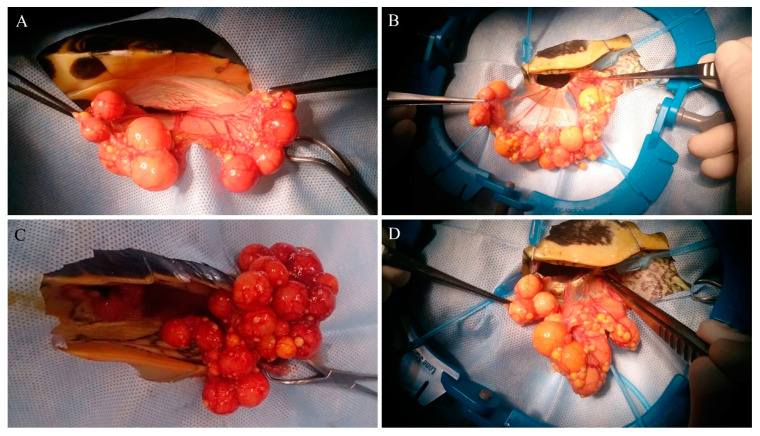
(**A**): Exteriorization of the left ovary from the left premoral fossa with the patient in right lateral recumbency. (**B**): Right ovary from the right premoral fossa with the patient in dorsal recumbency. (**C**): Right ovary from the left premoral fossa with the patient in right lateral recumbency. (**D**): Left ovary from the right premoral fossa with the patient in dorsal recumbency.

Once the ovary was identified, the avascular interfollicular connective tissue was grasped with straight Debakey forceps and gentle traction was applied to completely exteriorize the ovary from the breach and expose vascularization (Figure 2A,B). The ovarian vessels were ligated with stainless steel surgical ligation clips (Ligature Clip, Aesculap AG, Tuttlingen, Germany), and the mesovarium was transected with scissors. After resection of the ovary, the mesovarium was replaced inside the coelom, and an endoscopic inspection of the cavity was performed to verify hemostasis and check for residual follicles. For both groups, the ovary resection time was recorded from first grasping the interfollicular tissue to the endoscopic confirmation of total resection.

After the first ovary resection, the coelom was endoscopically inspected to identify the contralateral ovary. When possible, operative atraumatic forceps were used to grasp a portion of interfollicular tissue, and under endoscopic visualization, to apply gentle traction to it, drawing it to the incision and completely exteriorizing the ovary (Figure 2C,D) in order to perform the resection from the same surgical incision, in the same way as described above. For all groups, the second ovary resection time was recorded as described above.

The coelomic membrane and muscle aponeurosis were closed together with a 3.0 polydioxanon (PDS II, Ethicon, Johnson & Johnson, Pomezia, Italy) continuous suture pattern. The skin was closed with an everting horizontal mattress suture of the same material and covered with a thin layer of cyanoacrylate glue (Leukosan Adhesive, BSN Medical S.r.l., Agrate Brianza, Italy).

If extraction and resection of the second ovary were not feasible from a single surgical incision, access to the contralateral fossa was obtained, and surgery was performed as described above. In such cases, ease of identification would be recorded as 1 (Table 2).

Twenty minutes after closure, dexmedetomidine and midazolam were respectively reversed with an intravenous (IV) administration of atipamezole (Antisedan, Vetoquinol Italia S.r.l., Bertinoro, Italy) 1 mg/kg and flumazenil (Flumazenil, B. Braun Melsungen AG, Melsungen, Germany) 0.05 mg/kg.

A statistical analysis was performed using commercial statistical software (IBM SPSS statistics for Windows, Ver. 26.0). The Shapiro–Wilk test was applied to assess the normal distribution of the obtained values, and since the data were not normally distributed, a Mann–Whitney U test was applied to identify statistically significant differences (the null hypothesis being that no difference existed between the groups). Moreover, an ordinal logistic regression was applied to investigate the correlation between BW and ease of access to the coelom.

## 3. Results

Of the 27 turtles that met the inclusion criteria and underwent the procedure, three were excluded from the study after coelomic inspection: one animal had diffused coelomitis with hepatic granulomas, and two others revealed the presence of non-calcified eggs in the oviducts, which were not detected on the preoperative radiographic survey.

Regardless of the type of recumbency, the surgical access through the prefemoral fossa was easily achieved; this phase required less than 2 min (score 4 and 5), except in three cases (Table 3). No statistically significant differences were found between the scores describing the access to the coelom in both recumbencies, using a Mann–Whitney U test (*p* = 0.92). The median score of surgical access obtained in dorsal recumbency was 4 with an interquartile range (IQR) of 1, and 4.5 with an IQR of 1 for lateral recumbency.

The median BW was 1480 g (SD ± 382.5 g; range 925–2200 g). A positive correlation was identified between BW and the access score by analyzing the data with ordinal logistic regression. As BW increased, the score decreased; the weight was therefore predictive of the difficulty in creating access to the coelom (Table 4).

In all cases, the ovary on the same side as the surgical incision could be directly identified without endoscopic assistance, while the contralateral ovary was more difficult to locate in both recumbencies, with its visualization often only being possible through the membrane, requiring more manipulation of the surrounding structures. Animals in lateral recumbency had a median score of 2.5 with an IQR of 2, while in dorsal recumbency a median score of 3.5 with an IQR of 3 was recorded. Differences between the recumbencies were not statistically significant, as assessed using a Mann–Whitney U test (*p* = 0.23).

Regarding the removal of the ovary ipsilateral to the surgical access, from first grasping the interfollicular tissue to the complete resection of the mesovarium, it took a median time of 6.5 min (an IQR of 6) in lateral recumbency and 7 min (an IQR of 6) in dorsal recumbency (Table 5). The contralateral ovary’s median time of asportation was 3.5 min (an IQR of 7) in lateral recumbency and 4 min (an IQR of 5) in dorsal recumbency; the time of removal of the second ovary was not calculated for those animals that required a bilateral approach (Table 5). The median surgery time of the whole procedure was 39 min (an IQR of 19). In dorsal recumbency, the median surgery time was 39 min (an IQR of 29), and in lateral recumbency it was 39.5 min (an IQR of 15). No statistically significant difference was found between the two types of recumbency regarding the first ovary removal time (*p* = 0.52), second ovary removal time (*p* = 0.72), or total surgical time (*p* = 0.47), analyzed with the Mann–Whitney U test (Table 5 and Table 6).

In three cases (12.5%; one case in dorsal recumbency and two in lateral recumbency), a change in recumbency was necessary to remove the second gonad, it being necessary to switch the surgical access to the contralateral prefemoral fossa to carry out the surgery.

## 4. Discussion

No difference was observed between lateral and dorsal recumbency regarding ease of access to the coelom, ease of identification of the contralateral ovary, surgical times, or necessity to select a bilateral access. A single incision allowed the removal of both ovaries in 87.5% of the subjects, while the remaining animals needed a second incision due to a shorter and less extensible mesovarium, probably caused by seasonally related factors, which did not allow full exposure of the contralateral ovary. A positive correlation between BW and difficulty accessing the coelom was found.

Animals were selected from a limited geographical area to minimize environment-related variables, in order to have a more homogeneous population regarding ovarian cycles. Further distinction regarding the subspecies (*Trachemys scripta scripta*, *T. s. elegans*, *T. s. troostii*) was not considered necessary for the present work.

To assess the ease of access to the coelom cavity, a previously published scoring system [13] was modified and applied in our study; modifications were necessary since, in the original paper, access to the coelom was gained with the placement of an operating sheath and the replacement of the obturator with a telescope, thus requiring more time compared to a plain surgical access technique, such as the one applied in the present work. Out of 24 animals, 21 had excellent-to-good ease of access (requiring less than 2 min), and the other three required up to 4 min (a score of 3). No statistical difference was found between the two groups, but a positive correlation was found between the BW of the animals and the difficulty to access the coelom. This can be explained by the fact that bigger animals have deeper prefemoral fossae (thus making tissue and instrument manipulation more difficult), and more abundance of subcutaneous tissues that need to be delicately dissected in order to avoid iatrogenic trauma to the coelomic organs. The left approach with right lateral recumbency and the right approach with dorsal recumbency were selected because they are more comfortable for a right-handed surgeon.

Ease of identification of the ovary opposite to the surgical incision was assessed by adapting a previously published scoring system [13]; in the original paper, the scoring system was used to assess ease of identification of the coelomic organs, while in this case, the authors felt the necessity to slightly modify it in order to better adapt it to a large and mobile organ such as the chelonian mature ovary.

The mean total surgery time was consistent with previously published data [2,5,6,7,8,9], with no significant difference between the two recumbencies. In both recumbencies, removal of the first ovary took longer than the second, probably because the removal of the first ovary made more room inside the coelom, making it easier to completely exteriorize the second.

In 21 out of 24 animals (87.5%), bilateral oophorectomy could be achieved from a single incision. The three animals that required a bilateral approach underwent surgery in late August and early September. In these subjects, both ovaries were found to be smaller, and despite the fact that the contralateral ovary could be nonetheless easily identified and located, it could not be completely exteriorized due to the shorter length and lower extensibility of the mesovarium—thus the need for a second incision. The total surgery times in these subjects were 71, 40, and 68 min, and these were not excluded from the statistical analysis. A decrease in the mass of ovarian follicles from July to October has been described in wild specimens of *T. scripta elegans* in Southern Europe [15], but in our experience, such a decrease was observed only in late summer. This could suggest that in humid continental climates such as Po Valley, the best time to perform elective oophorectomy in this species is from middle–late spring to mid-August.

Further studies may be useful to also ensure the validity of our findings in other aquatic species with different anatomical features—for example, the Florida red-bellied cooter (*Pseudemys nelsoni*, which displays a high dome-shaped shell) or box turtles (*Terrapene* spp., *Cuora* spp.).

## 5. Conclusions

The results of this study are consistent with the previously published statement that the recumbency for prefemoral ovariectomy in pond sliders can be indifferently chosen based on the preference of the surgeon, on the animal’s pathophysiologiacal status (e.g., necessity for unilateral oophorectomy), or on the necessity to perform other procedures concurrently with ovariectomy (e.g., investigation of right or left liver lobe).

## Figures and Tables

**Table 1:** Scoring system for ease of access to the coelom, modified from Divers et al., 2010.

Score		Time (Minutes)
5	Excellent	<1
4	Good	1 to 2
3	Satisfactory	>2 to 4
2	Difficult	>4 to 5
1	Very difficult	>5

**Table 2:** Scoring system for ease of identification of the ovary opposite to the surgical incision, modified from Divers et al., 2010.

Score	Identification
5	Immediately recognizable
4	Recognizable through a membrane (e.g., mesentery, mesovarium)
3	Not immediately recognizable, need for minor visceral manipulation
2	Not immediately recognizable, need for major visceral manipulation
1	Not recognizable, impossible to exteriorize

**Table 3:** The score record regarding ease of access to the coelom and ease of identification of the ovary opposite to the surgical incision.

Recumbency	Ease of Access (Score)	Number of Subjects/12	Identification Second Ovary (Score)	Number of Subjects/12
Lateral	1	0	1 *	2
	2	0	2	4
	3	2	3	3
	4	4	4	2
	5	6	5	1
Dorsal	1	0	1*	1
	2	0	2	3
	3	1	3	2
	4	6	4	3
	5	5	5	3

* In all cases that scored 1 in ease of identification of the second ovary, the ovary was identified but impossible to exteriorize from the first surgical breach.

**Table 4:** Correlation between the access score and weight.

Coelom Access Score	Mean BW (g)	Standard Deviation
3	1788.3	357.5
4	1592.5	489
5	1206.3	315.4
Total	1440	447.2
*p* value *	0.013	

BW = body weight. * analyzed with ordinal logistic regression.

**Table 5:** Surgical timing of ovary removal.

		Lateral Recumbency	Dorsal Recumbency	Total
First ovary removal time	Median (minutes)	6.5	7.0	6.5
	IQR	6.0	6.0	6.0
	*p* value *	0.52		
Second ovary removal time **	Median (minutes)	3.5	4.0	4.0
	IQR	7.0	5.0	6.0
	*p* value *	0.72		

IQR= interquartile range. * analyzed with Mann–Whitney U test; ** animals that required a bilateral approach were not included in the calculation.

**Table 6:** Total surgical timing.

	Lateral Recumbency	Dorsal Recumbency	Total
Median (minutes)	39.5	39.0	39.0
IQR	15.0	29.0	19.0
*p* value *	0.47		

* analyzed with Mann–Whitney U test.
